# Comparison of ZnO Nanoparticles Prepared by Precipitation and Combustion for UV and Sunlight-Driven Photocatalytic Degradation of Methylene Blue

**DOI:** 10.3390/toxics11030266

**Published:** 2023-03-14

**Authors:** Sucheewan Krobthong, Tipawan Rungsawang, Sutthipoj Wongrerkdee

**Affiliations:** Department of Physical and Material Sciences, Faculty of Liberal Arts and Science, Kasetsart University Kamphaeng Saen Campus, Kamphaeng Saen, Nakhon Pathom 73140, Thailand; faasscw@ku.ac.th (S.K.); tipawan.r@ku.th (T.R.)

**Keywords:** ZnO, precipitation, combustion, photocatalyst, methylene blue

## Abstract

ZnO nanoparticles (NPs) were comparatively synthesized via precipitation and combustion techniques. The ZnO NPs synthesized via precipitation and combustion exhibited similar polycrystalline hexagonal wurtzite structures. The large crystal sizes of ZnO NPs were obtained from the ZnO precipitation in comparison with those from the ZnO combustion, while the particle sizes were in the same range. The functional analysis implied that the ZnO structures had surface defects. Moreover, absorbance measurement showed the same absorbance range in ultraviolet light. In the photocatalytic degradation of methylene blue, ZnO precipitation exhibited higher degradation performance than ZnO combustion. This was attributed to the larger crystal sizes of ZnO NPs, which provided an enduring carrier movement at semiconductor surfaces and reduced electron-hole recombination. Thus, the crystallinity of ZnO NPs can be considered an important factor in photocatalytic activity. Furthermore, precipitation is an interesting synthesizing method for preparing ZnO NPs with large crystal sizes.

## 1. Introduction

Synthetic dyes are a concern in wastewater because they can have negative impacts on the environment and human health [1]. Some dyes contain harmful chemicals that can be harmful to fish and other aquatic organisms when they are present at high concentrations in the water. Many dyes are resistant to traditional wastewater treatment processes [2], which means that they can pass through treatment plants and appear in rivers, lakes, and other bodies of water. Thus, wastewater containing synthetic dyes is a consideration for degrading and reducing toxic chemicals. The photocatalytic degradation of contaminating dyes is considered an interesting process for wastewater treatment because it can break dye molecules into smaller and less toxic compounds [3]. Photocatalytic degradation is a simple method of wastewater treatment that can be scaled up, such as in its applications in agricultural farms, industrial estates, and ranches [4]. The photocatalytic degradation of dyes requires semiconductor materials and processing in the presence of light [5]. There are several materials that can be employed as photocatalysts, including titanium dioxide (TiO_2_), tin oxide (SnO_2_), and zinc oxide (ZnO) [6]. These popular materials are often studied for their photocatalytic properties due to their wide bandgap energy (E_g_) and high surface area resulting in efficient light absorption and enhanced catalytic activity, respectively. Among these, ZnO is a well-known multifunctional-semiconductor material that has been widely investigated for its usage as a photocatalyst [7] because of its good photocatalytic activity, low-cost material, and non-toxicity [8,9,10]. Importantly, suitable synthesis methods have been studied, such as microwave synthesis, laser-ablation, hydrothermal, sol-gel, precipitation, and combustion [11,12,13,14,15].

Precipitation and combustion are interesting methods because they are low-cost and rapid processes. ZnO was synthesized for precipitation using a variety of zinc sources, including Zn(CH_3_COO)_2_·2H_2_O and Zn(NO_3_)_2_·6H_2_O [16,17,18]. These ZnOs had hexagonal wurtzite structures with a variety of morphologies. Absorbance spectra cover both Ultraviolet (UV) and visible regions, suggesting that they can be activated by UV or visible light. The ZnO nanostructures were demonstrated to be interesting photocatalysts, and they showed acceptable photocatalytic degradation of methylene blue (MB). In studies of combustion methods, synthesized ZnO NPs have been presented for use as photocatalysts [19,20,21]. Although the ZnO NPs were prepared at different temperatures, there were similar hexagonal wurtzites. However, surface impurities or surface defects were obtained more frequently than those of ZnO prepared by precipitation. For the photocatalytic test, it presented a potential for degrading dyes and several toxic molecules.

The mentioned literature has proven that ZnO is an interesting photocatalyst to degrade dyes and pesticides under UV or sunlight exposure. However, ZnO NPs synthesized by precipitation and combustion have not been directly compared in terms of photocatalyst application. Thus, the present work aims to directly compare the structural and optical properties and photocatalytic activity of ZnO NPs prepared by precipitation and combustion. These methods were selected to investigate the effect of the preparation method on the characteristics of ZnO NPs and their photocatalytic activity. It is well-known that small particle sizes play an important role in offering large surfaces for photocatalytic activity [22]. The combustion method is aimed at producing small ZnO NPs to observe their effect on photocatalytic activity. On the other hand, the high crystallinity of ZnO NPs synthesized by the precipitation method is considered to offer long durations of carrier transport at the ZnO surface, which potentially increases activity duration at the surface. Therefore, the methods were comparatively investigated.

## 2. Materials and Methods

**Figure 1:**
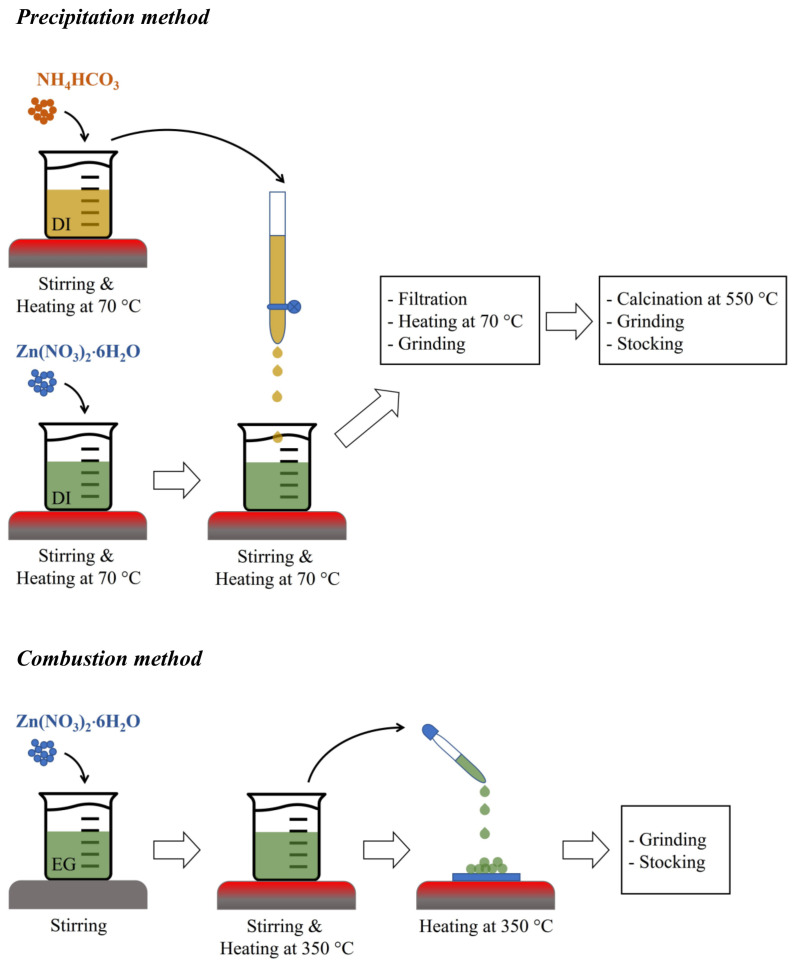
The illustration of ZnO preparation via precipitation and combustion methods.

ZnO NPs were synthesized via the precipitation process by mixing a 0.2 M aqueous solution of Zn(NO_3_)_2_·6H_2_O with a 0.4 M aqueous solution of NH_4_HCO_3_ in DI water. The starting aqueous solutions were separately prepared, stirred, and heated at 70 °C for 1 h. Then, the NH_4_HCO_3_ solution was added dropwise into the Zn(NO_3_)_2_·6H_2_O solution at a dropping rate of 2 mL/min [23]. The mixed solution was heated and stirred for an additional hour and then cooled down. Afterward, the obtained white product was filtered, heated at 70 °C for 3 h, and ground. The obtained product was then calcined at 550 °C for 6 h and ground again to obtain “ZnO precipitation”. For the combustion process, a 1 M aqueous solution of Zn(NO_3_)_2_·6H_2_O was prepared by dissolving the compound in 20 mL of ethylene glycol (EG) and stirring for 1 h at room temperature [11]. The resulting aqueous solution was then heated to 350 °C for 20 min on a hotplate. Meanwhile, cleaned glass slides were heated to the same temperature on another hotplate to serve as substrates for the combustion process. The Zn(NO_3_)_2_·6H_2_O solution was then dropped onto the heated substrates and allowed to undergo combustion for 20 min, followed by cooling down to room temperature. The products of the combustion were scratched from the substrates. The resulting material was identified as “ZnO combustion” after being ground. The precipitation and combustion methods are briefly illustrated in Figure 1.

The obtained ZnO NPs, ZnO precipitation, and ZnO combustion, were characterized by a Scanning Electron Microscope (SEM, JSM-6610 LV, JOEL, Bangkok, Thailand) for morphological observation. Microstructural analysis was conducted using Transmission Electron Microscopy (TEM, TECNAI G^2^ 20 S-TWIN, FEI, Nakhon Ratchasima, Thailand). Particle size distribution in DI water was analyzed using Dynamic Light Scattering (DLS, Beckman Coulter, Delsa Nano C, Bangkok, Thailand). XRD (D8 Advance, Bruker, Khon Kaen, Thailand), which was conducted to analyze the crystalline structures of the ZnO NPs. Selected Area Electron Diffraction (SAED) was performed to analyze the crystalline structure. Raman spectrometer (Thermo Scientific, DXR SmartRaman, Bangkok, Thailand) was used to investigate the vibrational nature of the ZnO NPs. Fourier Transform Infrared Spectrophotometry (FTIR, Spectrum Two, PerkinElmer, Nakhon Pathom, Thailand) was employed to examine the surface functional groups present on the NPs. A UV–VIS–NIR spectrophotometer (Hitachi, UH1450, Bangkok, Thailand) was utilized to monitor the absorbance spectra, and a spectrofluorometer was used to observe the fluorescence spectra.

In the photocatalytic activity test, the produced ZnO NPs via precipitation and combustion were utilized as photocatalysts for MB degradation. The starting MB solution was prepared by dissolving 5 mg of MB in 1 L of DI water (pH ~ 6.93). It was then stirred in the dark for 1 h. The solution was divided into two beakers, each containing 100 mL. Each beaker was loaded with a portion of 0.1 g of the ZnO NPs and stirred for 10 min in the dark. The mixtures were then placed in the photocatalytic activity station and left for 30 min in the dark to reach adsorption-desorption equilibrium. Afterward, the MB solution containing ZnO NPs was irradiated with UV light (Philips TUV 30W, 3 lamps, Nakhon Pathom, Thailand) to activate the photocatalytic activity. The solutions were sampled to measure absorbance using a UV–VIS spectrophotometer (BioMate 160, Nakhon Pathom, Thailand) and evaluate the photocatalytic activity. Furthermore, the photocatalytic degradation of MB was tested in natural sunlight to demonstrate its effectiveness and usability.

## 3. Results and Discussion

**Figure 2:**
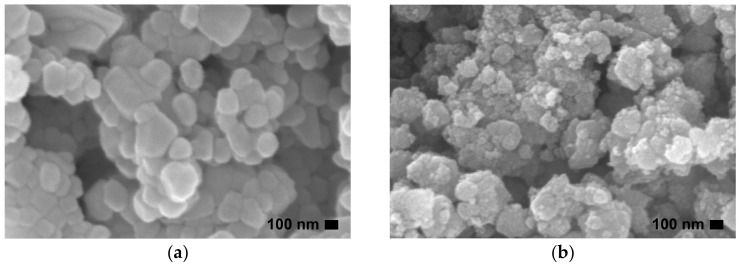
SEM morphological images of ZnO prepared by (**a**) precipitation and (**b**) combustion.

**Figure 3:**
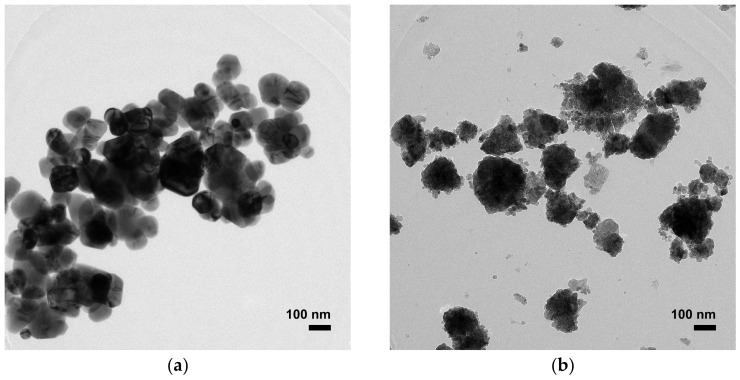
TEM microstructural images of ZnO prepared by (**a**) precipitation and (**b**) combustion.

**Figure 4:**
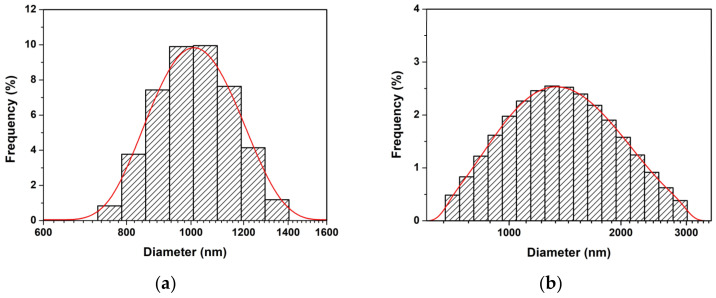
Hydrodynamic diameter of aggregates of ZnO NPs prepared by (**a**) precipitation and (**b**) combustion.

The morphology and microstructure of prepared ZnO NPs are presented in Figure 2 and Figure 3, respectively. The large NP sizes appeared for ZnO precipitation, while the aggregations of numerous NPs were observed for the ZnO combustion. This refers to the fact that ZnO precipitation has a lower NP boundary than ZnO combustion. The large NP sizes of ZnO precipitation can provide long paths for carrier (electron-hole pairs) movement at the ZnO surface [4]. Moreover, the large NP sizes also reduce recombination due to carrier-boundary collision. The different structures of ZnO NPs prepared by precipitation and combustion in this study might be due to differences in reaction process, temperature, and reaction time. DLS measurements, shown in Figure 4, were used to investigate the particle sizes of ZnO NPs prepared by precipitation and combustion in an indirect manner. The particle sizes of the ZnO precipitation were found in terms of hydrodynamic diameters to be 700–1400 nm, while the diameters of the ZnO combustion were 700–3000 nm. The average sizes of ZnO precipitation and ZnO combustion were 1022 and 1462 nm, respectively. It is noted that the DLS results present larger particle sizes of ZnO NPs than SEM and TEM images. This could be an agglomeration of NPs in DI water, which results in a larger hydrodynamic size detection. The large distribution for ZnO combustion could be attributed to NP impurities, which cause low stability in DI water and result in stronger agglomeration. Although the ranges are different, there is a similar nature to the aggregates of ZnO NPs. The aggregation effect might reduce the surface area of ZnO NPs, which would slow down the photocatalytic activity.

**Figure 5:**
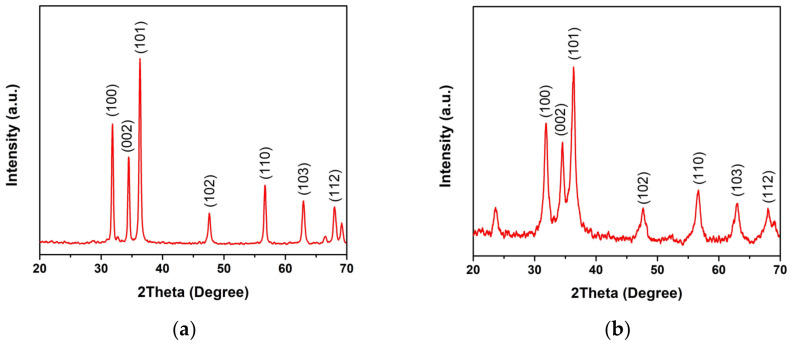
XRD patterns of ZnO NPs prepared by (**a**) precipitation and (**b**) combustion.

**Figure 6:**
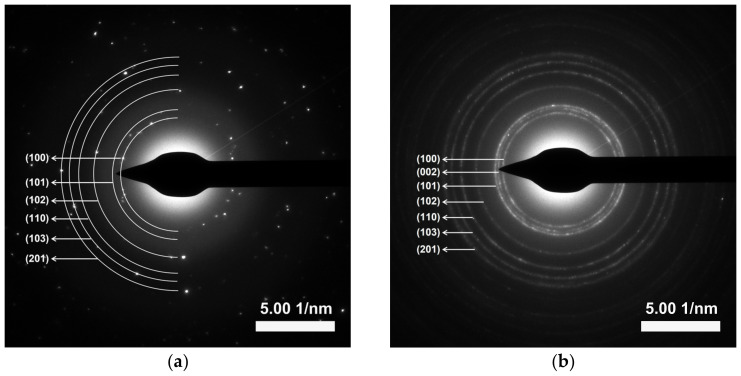
SAED ring-like patterns of ZnO NPs prepared by (**a**) precipitation and (**b**) combustion.

The crystal structure determined by XRD patterns is presented in Figure 5. The XRD patterns are well-matched with standard JCPDS no. 36-1451 [24,25], which indicates the hexagonal wurtzite structure of ZnO. The appeared additional peak at 2θ~23.5° for ZnO combustion can refer to the orthorhombic phase of Zn(OH)_2_ (JCPDS no. 01-071-2215) caused by the incomplete reaction [11]. The XRD peaks for ZnO precipitation were narrower than those for ZnO combustion, indicating that the crystal size of ZnO precipitation is larger than that of the ZnO combustion. The crystal size (D) was calculated using the Scherrer equation (Equation (1)) [26,27,28]:

D = kλ/βcosθ(1)

where k and λ are the shape factor of XRD measurement (0.89) and the wavelength of the X-ray source (1.5406 Å), respectively. The β and θ are the full width at half maximum (FWHM) and the diffraction angle for each plane, respectively. The crystal sizes calculated from the three major peaks of (100), (002), and (101) planes were 45.5 ± 10.0 and 10.4 ± 2.6 nm for ZnO precipitation and ZnO combustion, respectively, in agreement with the SEM results. The larger crystal size for ZnO precipitation reflects better crystallization in comparison with ZnO combustion because the precipitation process provides a longer chemical reaction time for crystallization. On the other hand, the reaction can be limited by the short duration of the combustion process. The SAED patterns (Figure 6) exhibited similar ring-like patterns for ZnO precipitation and ZnO combustion, indicating polycrystalline structures due to random phase growth [23].

**Figure 7:**
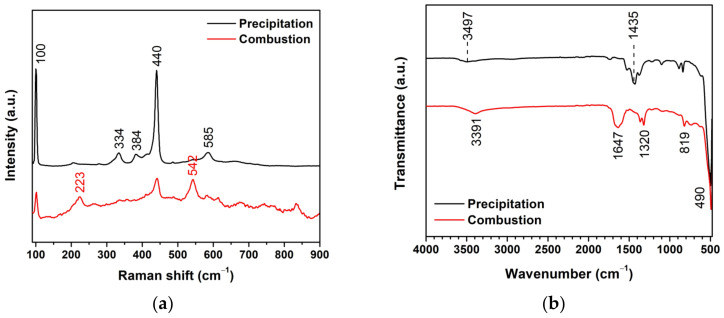
Vibrational analysis of ZnO NPs prepared by precipitation and combustion: (**a**) Raman shift and (**b**) FTIR spectra.

Figure 7a depicts the Raman shift for vibrational analysis of ZnO NPs. The ZnO NPs revealed dominant peaks at 100 and 440 cm^−1^, which assigned the E_2_ (low) and E_2_ (high) modes, respectively [29,30]. The E_2_ (low) vibration represented the zinc crystal lattice in ZnO, while the E_2_ (high) vibration represented the oxygen vibration. The peaks at 334, 384, 542, and 585 cm^−1^ were assigned to the E_2_ (high)–E_2_ (low), A_1_ (TO), A_1_ (LO), and E_1_ (LO) modes, respectively [31]. The peak at 223 could be assigned to 2TA or 2E_2_ (low) modes. [30,32]. These peaks indicate the disordered structures for both ZnO NPs prepared by precipitation and combustion. Moreover, the E_2_(low) and E_2_(high) modes show low intensity compared with the ZnO precipitation. These behaviors suggest the presence of oxygen defects in the ZnO combustion. In Figure 7b, FTIR spectra in the range of 4000–450 cm^−1^ were analyzed to investigate functional groups of the ZnO NPs. The peaks at 490 cm^−1^ contributed to the ZnO structure, due to the Zn-O vibration [33]. The peaks at 819 and 1647 cm^−1^ corresponded to C=O stretching [11]. The peaks at 1320 and 1435 cm^−1^ corresponded to C-O stretching and C-H blending, respectively [11]. The peaks at 3391 and 3497 cm^−1^ were assigned to the O-H stretching. The appearance of these peaks implies the presence of residuals.

**Figure 8:**
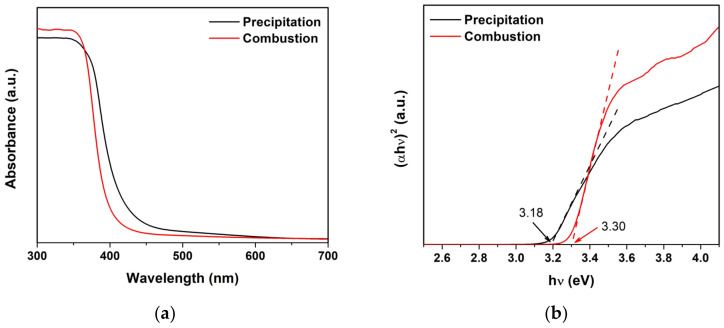

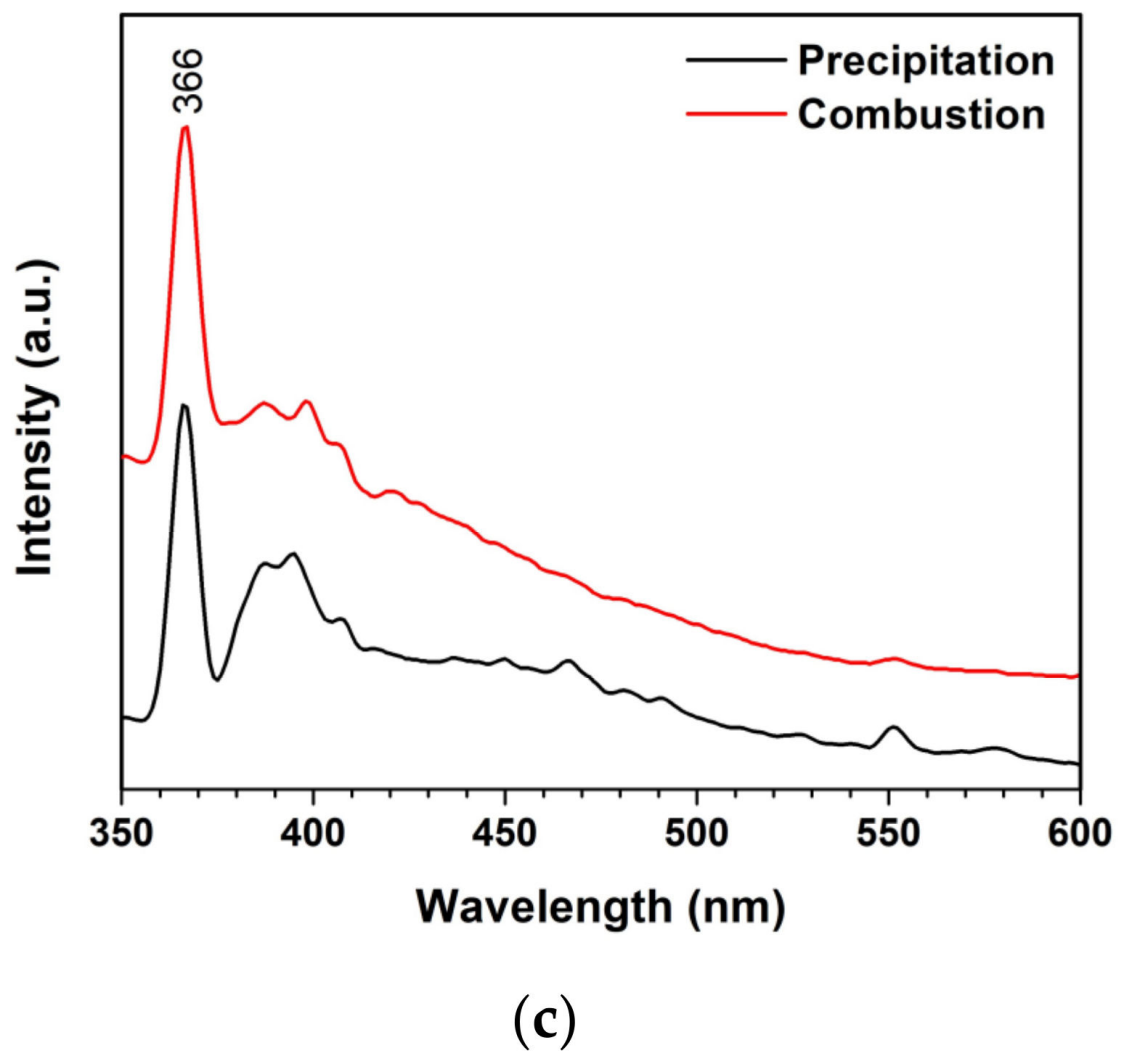
(**a**) absorbance, (**b**) band gap energy analysis, and (**c**) fluorescence spectra of ZnO NPs.

The optical absorbance was measured (Figure 8a) to survey the energy absorption of the ZnO NPs. The band gap energy (E_g_) of ZnO was estimated using Equation (2) [11,17]:

(αhν)^2^ = A(hν − E_g_) (2)

where α, h, ν, and A are the absorption coefficient, Planck’s constant, frequency, and constant, respectively. The (αhν)^2^ vs. hν curve, Figure 8b, was used to calculate the E_g_ value. E_g_ values of the ZnO precipitation and the ZnO combustion are 3.18 and 3.30 eV, respectively. The difference in E_g_ for ZnO precipitation and ZnO combustion might be due to structural disorders, chemical defects, and impurities. The E_g_ values correspond to the range of the UV region, which indicates that these ZnO NPs can be utilized as photocatalysts under UV irradiation. Note that the lower E_g_ for the ZnO precipitation refers to the fact that it can be excited with lower incident energy in comparison with the ZnO combustion. The fluorescence spectra of ZnO NPs excited at 325 nm are shown in Figure 8c. The ZnO precipitation and the ZnO combustion present a similar emission peak in the UV region at 366 nm. The behavior might be electron-hole recombination, referring to the nature of ZnO. However, surface defects are detected according to the minor broad peaks at around 387–407 nm.

**Figure 9:**
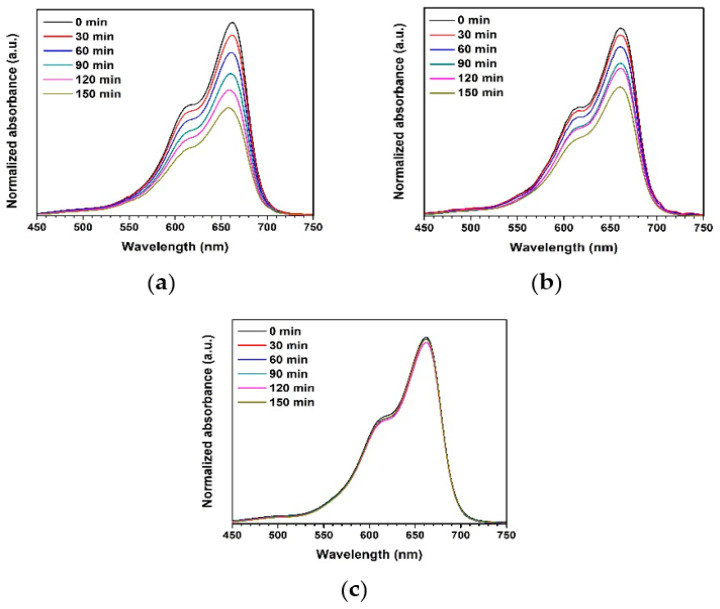
Normalized absorbance of MB solution under UV light using different photocatalysts: (**a**) ZnO precipitation, (**b**) ZnO combustion, and (**c**) blank.

The ZnO precipitation and the ZnO combustion were preliminarily employed to degrade MB present in water under UV irradiation for investigating the photocatalytic degradation performance. The measured absorbance of the MB solution is shown in Figure 9. The absorbance decreased as the UV irradiation time increased, which indicates more degraded MB molecules for the longer irradiation periods. It is seen that the absorbance of the MB solution containing the ZnO precipitation decreased more in comparison with the solution containing the ZnO combustion. As a note on the degradation of MB under UV light without photocatalyst (blank), the absorbance of MB showed a non-significant change, indicating low degradation activity in the absence of a photocatalyst.

**Figure 10:**
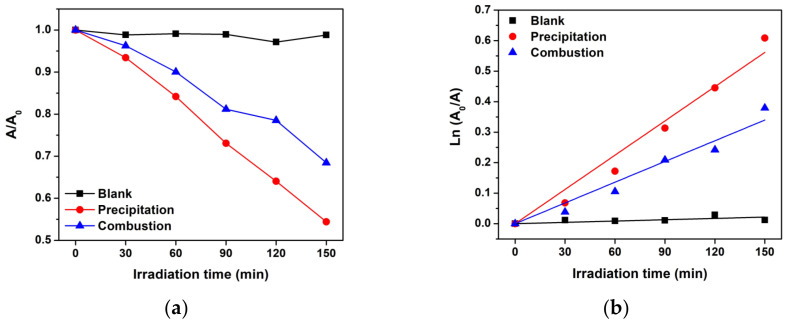
Photocatalytic performance of MB under UV light: (**a**) plot of A/A_0_ analysis, and (**b**) apparent degradation rate constant analysis.

As shown in Figure 10a, the interval to the initial absorbance (A/A_0_) ratio of MB was plotted to observe the change in MB after photocatalytic activity. When compared to ZnO combustion, the A/A_0_ of MB had a significantly lower value for ZnO precipitation. For a better comparison, the apparent degradation rate constant (k_app_) was analyzed using Equation (3) [9,28]:

ln (A_0_/A) = k_app_t (3)

where t is the irradiation time. The k_app_ was estimated from the slope of Figure 10b and listed in Table 1. The ZnO precipitation showed higher k_app_ values for the photocatalytic degradation of MB than for that of the ZnO combustion around 1.6 times. The result corresponds with the larger crystal sizes of the ZnO precipitation compared to those of the ZnO combustion.

**Figure 11:**
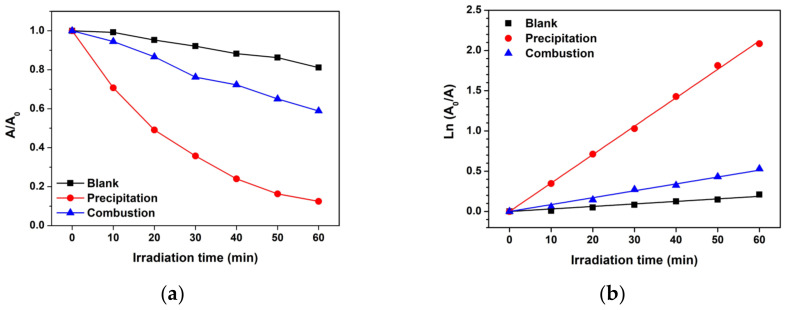
Photocatalytic performance of MB degradation under natural sunlight: (**a**) plot of A/A_0_ analysis and (**b**) apparent degradation rate constant analysis.

The application of ZnO photocatalysts was further investigated to degrade MB under natural sunlight irradiation (average sunlight intensity of 933.3 ± 70.8 W/m^2^) in order to demonstrate their effectiveness and usability. The absorbance of MB was monitored and analyzed as shown in Figure 11. The A/A_0_ ratio rapidly decreased for the ZnO precipitation, suggesting that the ZnO precipitation and the ZnO combustion have a positive effect on the photocatalytic degradation of MB under sunlight activation. It should be noted that in the blank condition, MB decreased with increasing irradiation time. This indicates stronger photolysis of MB under sunlight compared with UV light. Moreover, the k_app_ value reveals that the ZnO precipitation had the highest k_app_ value (Table 1). The result exhibits a similar trend to those under UV light. Thus, it can be confirmed that the ZnO precipitation is more effective in degrading MB compared to the ZnO combustion. It can be interpreted that the ZnO prepared by precipitation has a greater photocatalytic performance in comparison with the ZnO prepared by combustion. This effect can be due to the large crystal sizes of the ZnO NPs. It is well-known that large crystal sizes can provide a long lifetime for carrier movement at semiconductor surfaces and reduce electron-hole recombination [4]. On the other hand, short lifetime and recombination can occur for the small crystal size because carriers may hit many boundaries during the movement leading to low carriers for photocatalytic activity. Thus, it can be concluded that the crystallinity of the ZnO NPs prepared by precipitation has an impact on the photocatalytic activity for degrading MB molecules in water. Furthermore, precipitation is an interesting synthesizing method for preparing ZnO NPs with large crystal sizes in comparison with the combustion method. This study demonstrates the acceptable performance of ZnO photocatalysts for wastewater treatment [34,35,36]. On the other hand, the photocatalytic activity of ZnO NPs prepared by combustion exhibited lower performance in comparison with precipitation. This behavior might be caused by the presence of defects such as structural disorders, chemical defects, and impurities in the ZnO structure. This leads to the inhibition of carrier movement at ZnO surfaces, which reduces the photocatalytic activity.

## 4. Conclusions

ZnO NPs were comparatively synthesized via precipitation and combustion methods. The ZnO NPs prepared by precipitation and combustion exhibited similar polycrystalline hexagonal wurtzite structures. Hydrodynamic sizes of ZnO precipitation and ZnO combustion were 1022 and 1462 nm, respectively. The large crystal sizes of ZnO NPs were obtained for the ZnO precipitation in comparison with those of the ZnO combustion. However, the functional analysis implied the presence of ZnO structures with surface defects. Moreover, the absorbance measurement showed the same absorbance range in the UV region. For photocatalytic degradation of MB, the ZnO precipitation showed higher degradation efficiencies and apparent degradation rates constant than the ZnO combustion. This was due to the large crystal sizes of the ZnO NPs, which provided an enduring carrier movement at semiconductor surfaces and reduced electron-hole recombination. Thus, the crystallinity of the ZnO NPs can be considered an important factor in photocatalytic activity. Furthermore, precipitation is an interesting synthesizing method for preparing ZnO NPs with large crystal sizes.

## Figures and Tables

**Table 1:** Apparent degradation rate constant for photocatalytic degradation of MB under UV and natural sunlight irradiation using ZnO NPs.

Photocatalyst	k_app_ (10^−3^ min^−1^)	
	UV	Sunlight
Blank	0.14	3.13
ZnO precipitation	3.74	35.31
ZnO combustion	2.27	8.56

## Data Availability

Not applicable.
