# Correction: Krobthong et al. Comparison of ZnO Nanoparticles Prepared by Precipitation and Combustion for UV and Sunlight-Driven Photocatalytic Degradation of Methylene Blue. *Toxics* 2023, *11*, 266

**DOI:** 10.3390/toxics11050436

**Published:** 2023-05-06

**Authors:** Sucheewan Krobthong, Tipawan Rungsawang, Sutthipoj Wongrerkdee

**Affiliations:** Department of Physical and Material Sciences, Faculty of Liberal Arts and Science, Kasetsart University Kamphaeng Saen Campus, Kamphaeng Saen, Nakhon Pathom 73140, Thailand

## Error in Figure

In the original publication [1], there was a mistake in Figure 9a,b as published. The position of Figure 9a,b was misplaced. The corrected position of Figure 9a,b appears below. The authors state that the scientific conclusions are unaffected. This correction was approved by the Academic Editor. The original publication has also been updated.

## Figures and Tables

**Figure 9:**
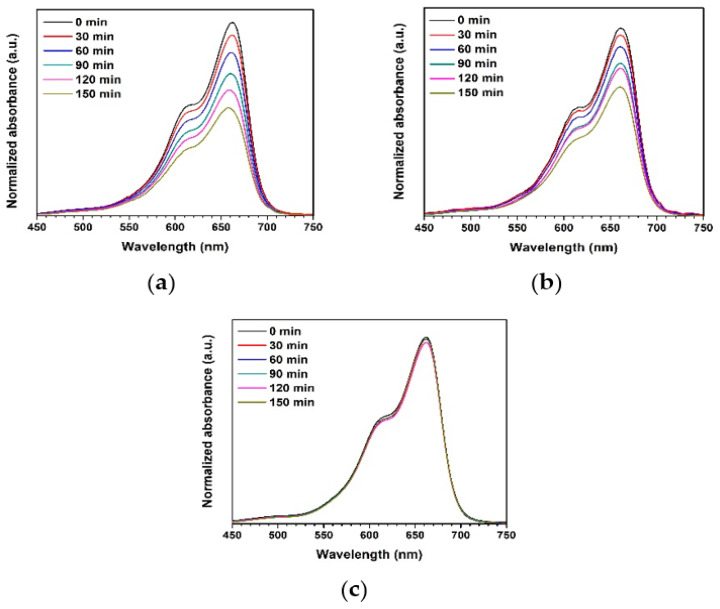
Normalized absorbance of MB solution under UV light using different photocatalysts: (**a**) ZnO precipitation, (**b**) ZnO combustion, and (**c**) blank.

## References

[1] Krobthong S., Rungsawang T., Wongrerkdee S. (2023). Comparison of ZnO Nanoparticles Prepared by Precipitation and Combustion for UV and Sunlight-Driven Photocatalytic Degradation of Methylene Blue. Toxics.

